# Expert views on high fat, salt and sugar food marketing policies to tackle obesity and improve dietary behaviours in the UK: a qualitative study

**DOI:** 10.1186/s12889-023-16821-2

**Published:** 2023-10-09

**Authors:** Shona Hilton, Caroline Vaczy, Christina Buckton, Chris Patterson, Marissa J. Smith

**Affiliations:** MRC/CSO Social and Public Health Sciences Unit, University of Glasgow, 99 Berkeley Square, Glasgow, G3 7HR UK

**Keywords:** Public health, Health promotion, High fat sugar and salt, Food marketing, United Kingdom, Obesity

## Abstract

**Background:**

There has been a lack of progress in reducing obesity in the United Kingdom (UK) despite Government strategies released over the last 30 years. These strategies, including the most recent publication from July 2020, have focused on childhood obesity and high fat, sugar and/or salt (HFSS) marketing restrictions, particularly broadcast advertising. In this study, we aimed to examine a range of expert views on the potential impact and the relative importance of such policies.

**Methods:**

Semi-structured interviews were conducted with 42 experts in policy (*n* = 19), industry (*n* = 10), and advocacy (*n* = 13) with an interest in obesity. The UK Government’s 2020 obesity strategy was used as a prompt to guide discussion on policy options. Qualitative thematic analysis was employed to answer the three research questions and themes were inductively coded within each research question. Data were also cross compared using matrix coding and a form of framework analysis to examine the views expressed by the different types of stakeholders.

**Results:**

Reactions to the July 2020 proposal were mixed among policy and advocacy stakeholders, while commercial stakeholders expressed disappointment. A main theme emerging in all groups was frustration with the policy process and wishing to see more clarity regarding restrictions and their implementation. There was an overall lack of trust that the government would carry out their proposed plan and agreement that a more comprehensive, multi-sector approach aimed at the underlying drivers of obesity would be most effective, with some stakeholders indicating that some of the proposed policies could make a difference if implemented robustly. On the theme of promoting healthier options, some stakeholders suggested lowering the prices of ‘healthy’ products and making them more accessible to combat regressivity. There was a potentially surprising level of agreement between policy/advocacy stakeholders and commercial stakeholders, although commercial stakeholders were more likely to advocate for collaboration between government and industry as well as voluntary industry measures.

**Conclusion:**

While HFSS marketing restrictions have a role to play and send a strong signal – provided they are implemented comprehensively – investment in these policies needs to be part of wider efforts to tackle the underlying drivers of obesity.

**Supplementary Information:**

The online version contains supplementary material available at 10.1186/s12889-023-16821-2.

## Introduction

Despite a 30-year history of the United Kingdom (UK) Government's obesity strategy, dating back to The Health of the Nation in 1992 [1] and spanning Conservative, Labour and Coalition Governments, there has been little progress in reducing the prevalence of overweight and obesity in the UK [2]. The majority of UK adults still live with overweight or with obesity (67% of men and 60% of women) and in 2019, 20% of year six children (aged 10–11) were classified as obese [3]. These high levels of overweight and obesity have serious consequences for population health. The 2021 National Food Strategy independent review identified that four out of the top five risk factors contributing to years lost to early death, ill-health and disability are diet related [4].

In recent years, the UK Government’s obesity strategies have emphasised dealing with childhood obesity, particularly through policies seeking to limit the exposure of children and young people to the marketing of foods high in fat, sugar and/or salt (HFSS) [5, 6]. This approach was extended to the whole population in the policy paper ‘Tackling obesity: empowering adults and children to live healthier lives’, published in July 2020, which included further restrictions on the marketing of HFSS products as a key component (Table 1) [7]. Some of the proposed policies, such as restrictions on in-store placement of HFSS foods, will only be enforced in England, however the geography of application is not relevant to this research.

**Table 1:** Tackling obesity: empowering adults and children to live healthier lives – actions and consultations contained within the policy paper (1)

• Introducing legislation to require large out-of-home food businesses, including restaurants, cafes and takeaways with more than 250 employees, to add calorie labels to the food they sell
• Legislating to end the promotion of HFSS foods by restricting volume promotions such as buy one get one free, and the placement of these foods in prominent locations intended to encourage purchasing, both online and in physical stores in England
• Banning the advertising of HFSS products being shown on TV and online before 9 pm
• Holding a consultation on how to introduce a total HFSS advertising restriction online

Table 1, extract from Tackling obesity: empowering adults and children to live healthier lives by Department of Health and Social Care

This increased willingness from the UK Government to take an interventionist approach at a population level, possibly driven by the Coronavirus (COVID-19) pandemic and the increased risk of worse health outcomes from contracting the virus for those living with obesity [8], was welcomed by health experts. However, a note of caution was sounded, with many highlighting that the announced policies were unlikely to be enough to tackle the underlying root causes of obesity, including stigma, poverty, and inequality [9–12].

Nevertheless, there is a growing body of evidence that commercial marketing activities can promote unhealthy purchasing and eating behaviour, including: advertising [13]; price promotions [14]; nutritional labelling [15]; and in-store placement schemes [16]. The regulation of commercial marketing activities is therefore important [17] and particularly warranted when there is a significant impact on the health of children and young people [18].

The UK Government reaffirmed its commitment to the 2020 Obesity strategy in the Queen’s speech at the state opening of Parliament in May 2021. Most recently, however, the Government has delayed the introduction of a 9 pm TV watershed for HFSS products and a restriction of paid-for HFSS advertising online [19], further highlighting the lack of follow-through seen previously in policy implementation [2].

Responses submitted by academics and researchers to the 2020 Obesity strategy have been published [9, 20], but there has not yet been a qualitative study on the reactions of stakeholders from a range of backgrounds. Now approaching three years after the publication of the strategy, momentum among policymakers regarding obesity has waned alongside delays in implementation of the proposed regulations [21], indicating the importance of continuing the conversation. The purpose of this study is to understand expert views on the relative importance of HFSS marketing restrictions to improve eating behaviour and ultimately reduce overweight and obesity prevalence in the UK.

### Research questions

RQ 1: How impactful could the HFSS marketing restriction elements of the UK Government’s 2020 obesity strategy be?

RQ 2: What other policy areas are important to consider when seeking to reduce obesity at a population level?

RQ3: How might Government policy improve the availability and promotion of ‘healthy’ options?

## Methods

This section closely follows the methodological approach described in the Cancer Research UK report “One Year On… Building on Bold Policy Ambitions: Stakeholder views on HFSS marketing restrictions and the next steps to help tackle obesity” [22]. Qualitative methods were used to elicit the views of a range of stakeholders with expertise in obesity policy, from three different perspectives. We recruited from the following groups: policy experts including policymakers, regulatory bodies, academics, and third-sector organisations; commercial experts including industry stakeholders from the food and drink industry and advertising and broadcasting industries; and advocacy experts including those who represent those experiencing stigma (e.g., for their weight or eating disorders), people who live with inequalities and children and young people.

### Sampling and recruitment

The experience of the research team, both Cancer Research UK (CRUK) and the University of Glasgow, was used to develop a list of target stakeholders from each of the three categories with an appropriate level of expertise in obesity policy relevant to their area of interest [23] (Table 2). While purposive sampling does not aim to obtain a representative sample of the distribution of perspectives in a population, it does allow for a wide range of relevant views, including unexpected or uncommon ones, to be represented and as such was appropriate to answer the research questions in this study [24].

**Table 2:** Areas of expertise of potential participants contacted in initial recruitment efforts

Stakeholder Type	Area of expertise
Policy experts	Regulatory bodies
	Policymakers
	Semi-official policymakers
	Third sector/charities
	Academics
Commercial experts	Food and drinks industry
	Trade associations
	Retail sector
	Advertising industry
	Social media influencers
	CRUK corporate partners
	Broadcasters and internet platforms
Advocacy experts	Stigma community
	Poverty and inequalities
	People who work with children and young people
	Food and health journalists

**Fig. 1:**
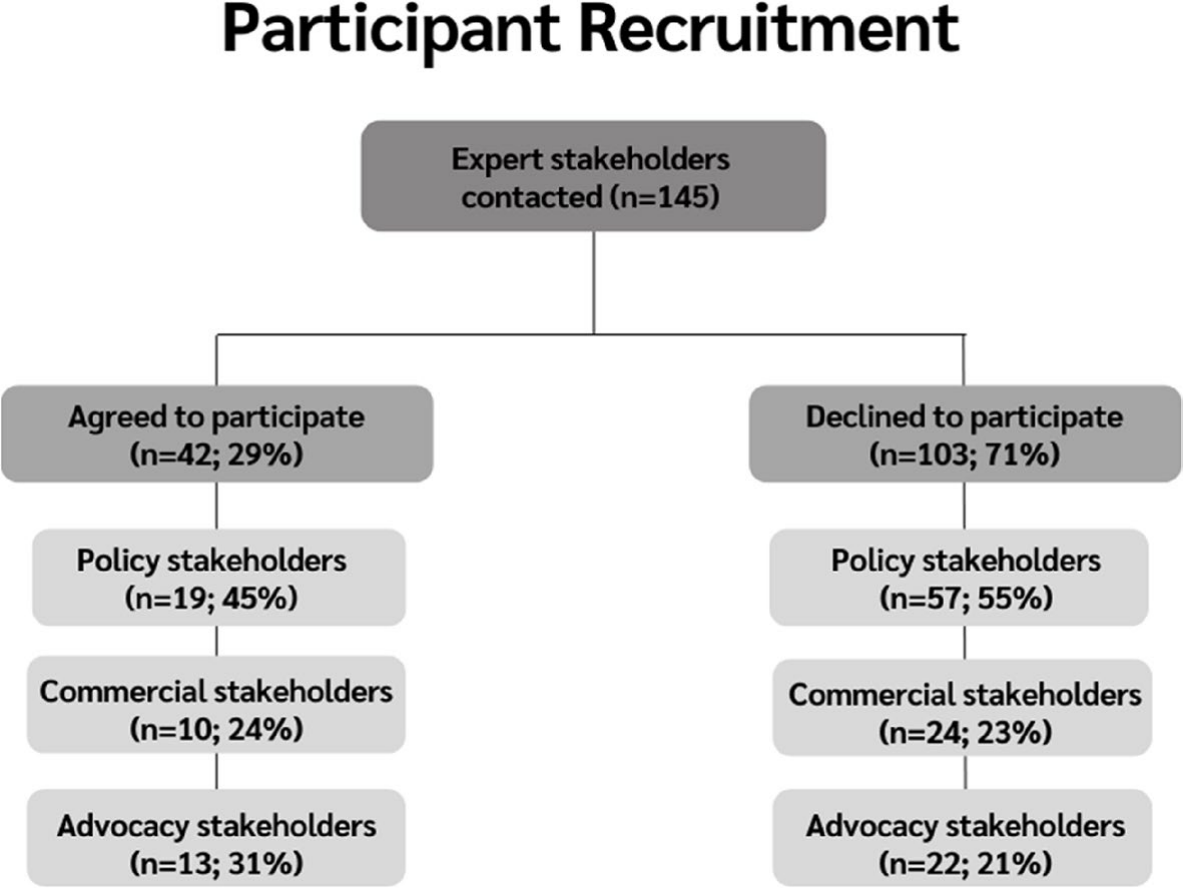
Recruitment process of expert stakeholders

Well-known individuals in target organisations or those prominent in their field with relevant policy expertise were identified (*n* = 145) and contacted by personal e-mail invitation from the senior author (SH, professor of public health and experienced qualitive researcher) to introduce the goals of the study. The research team’s experience and connections ensured a wide diversity of stakeholders participated in the interviews, representing a range of views. Professor SH’s reputation was helpful in securing participation from key individuals. Those who agreed to participate were provided with the study participant information sheet, privacy notice and consent form, which they signed and returned prior to being interviewed. Of the 145 individuals approached, 76 were policy stakeholders, 34 commercial stakeholders and 35 advocacy stakeholders (Fig. 1). Our final sample of 42 participants was sufficient to ensure data saturation was reached [23].

### Interview design

Semi-structured interviews, lasting between 45 and 60 min, were conducted by either CB or CP (both research assistants and experienced qualitive researchers, one male and one female) via Microsoft Teams video call and digitally recorded, between September and December 2020. Interviews were directed by an interview schedule (Appendix A) with sufficient flexibility to allow participants to focus on section(s) that reflected their own area of expertise or interests in obesity policy. Questions were split into four sections:

1. Exploration of views on the marketing restriction policies announced as part of the UK Government’s Obesity Strategy in July 2020 for implementation or further consultation (Table 1).
2. Exploration of views on a broader set of marketing restrictions and other policy options – based on suggestions proposed in the Chief Medical Officer (CMO) report on childhood obesity [17].
3. Exploration of views on key policy priorities, including those that could improve the availability and promotion of healthy food choices.
4. Exploration of contextual factors such as COVID-19 and Brexit.

Visual aids were used to prompt discussion of the policies under consideration in Sects. 1 and 2 (Appendix B). Policies were categorised by the 4Ps of the marketing mix: price, product, place and promotion [25].

### Data analysis

Audio recordings were transcribed verbatim and anonymised transcripts were imported into NVivo 12 for analysis. A draft coding frame was developed and tested by the research team using a process of double coding on a subset of five transcripts. In the first instance, deductive codes based on the interview topic guide were used to structure the data at a high level using a form of framework analysis driven by the research questions [26]. Using Braun and Clark’s six-phase method of thematic analysis [24], inductive themes were subsequently identified within each of the high-level deductive codes to capture different thematic aspects of participants’ experiences in a way that allowed direct comparisons across policies and stakeholder categories. The research team read and reread the transcripts to become familiar with the data, and then iteratively constructed a coding frame based on the topic to enable consistent organisation of relevant data. Stakeholders’ initial reactions to the UK Government’s policy announcement in July 2020 and their support for, or opposition to, each policy in the announcement were captured using attitudinal codes (Appendix D). Matrix coding allowed the research team to convert qualitative data into numerical data to examine stakeholder positions for each policy/theme and compare the arguments made by stakeholder type. Matrices are a central component of Gale et al.’s framework analysis, offering a way to ‘systematically reduce the data’ and compare responses between participants [26]. This method of analysis facilitated identification of common and atypical experiences and perspectives. Each transcript was imported into NVivo V12, coded independently, cross-checked and analysed by CB and CP. The diverse set of stakeholders and sample size of 42 allowed for data saturation to be reached. Contradictory cases and group dynamics were discussed, making use of transcripts and field notes. The interviewers reflected on their role as researchers, remained constantly aware of their position and took care not to introduce bias throughout the research. To further reduce bias, the researchers recorded the interviews and discussion groups and analysed them some time after they were completed, ensuring a more reflective viewpoint.

### Ethics

This study was granted ethical approval by the University of Glasgow, College of Social Sciences Research Ethics Committee. Approval was granted on 2 September 2020, application number 400190230.

## Findings

Of the 145 stakeholders approached, 42 participated in a semi-structured interview. Of these, 19 were policy experts, 10 commercial experts and 13 advocacy experts.

All interviews followed the same question structure, however individual interviewees tended to focus on elements that reflected their own areas of expertise and interests. For example, policy stakeholders were more likely to comment in detail on the specifics of individual policies, whereas commercial stakeholders tended to speak in more general terms. The following section will address each of the three research questions and the prominent themes that emerged in discussions of each.

### RQ 1: How impactful could the HFSS marketing restriction elements of the UK Government’s 2020 obesity strategy be?

#### Views on the proposal as a whole

##### Theme 1.1: overview of initial reactions to the July 2020 announcement

While individual interviewees clearly answered interview questions through the lens of their own policy interests there were some key underlying areas of agreement. Attitudinal analysis of interviewees’ initial reaction to the UK Government’s 2020 announcement revealed a mixed reaction from policy and advocacy stakeholders with most expressing cautious optimism:

*‘But in general, yeah, we were pleased to see acknowledgement through the obesity strategy of those environmental factors that contribute to excess weight and then subsequent poor health. And now we just need to see it implemented.’ (P15 policy)*

Some of these cautiously optimistic stakeholders expressed doubt as to whether the policies would be implemented in full while others considered the proposed steps to be too small. Commercial stakeholders were unsurprisingly disappointed:

*‘Disappointment, I think, because it’s the wrong policy, for a whole variety of different reasons. It won’t, I mean, not only will it not make any difference to childhood obesity, actually, I think it could make it worse in a few different ways.’ (P13 commercial)*

Many commercial stakeholders felt that the July proposal, particularly the restriction of HFSS food marketing and promotions, would target the wrong areas and therefore be ineffective.

##### Theme 1.2: a shared frustration with the policy process

The first common theme across all stakeholder types arose from a shared will to see proactive government action on obesity levels combined with a sense of frustration over the policy process in recent years and the persistent lack of detail on what specific HFSS marketing restrictions might entail, how they would be implemented, and which products they would apply to.

*‘Even lots of the consultations didn’t happen last time and the consultations that did happen, we haven’t had summaries of all of them yet so I’m not over optimistic that any of it is going to happen’ (S01 policy)*

Stakeholders highlighted many of the policies in the announcement had been subject to previous consultations with no outcome so far. Further advertising restrictions on TV and online [26], in-store promotion restrictions in the retail environment [27] and mandatory calorie labelling for the out-of-home sector [28] were mentioned as examples.

##### Theme 1.3: focussing on further HFSS marketing restrictions may represent a missed opportunity

Interviewees in all three groups welcomed a more interventionist approach to obesity policy from the UK Government, which some thought had been prompted by the COVID-19 pandemic, specifically Boris Johnson’s own personal experience. However, the focus on further HFSS restrictions without focusing on the whole diet was questioned by policy and advocacy stakeholders as it could divert attention away from dealing with more important drivers of obesity such as inequalities, social norms and the food environment more generally. Commercial stakeholders argued that the complexity of the food environment necessitates a holistic approach.

*‘I just think there’s logical inconsistencies in this plan, as there have been in others, which really miss some of the drivers of obesity we see in this country’ (S11 commercial)*

Despite these reservations, policy and advocacy stakeholders noted that the July announcement did send an important signal to the public and industry about the seriousness of the Government’s position on dealing with obesity, and that if the package of measures was implemented robustly it could make a difference.

*‘I think I would agree that if all of these policies were implemented as strictly as possible, so as in not watered down, I think that they could be a really good group of policies that cover quite a range of spaces.’ (S22 advocacy)*

##### Theme 1.4: policies would have more impact if positioned as being for the benefit of the whole population

All stakeholders were concerned that the framing of the strategy as being about obesity, particularly in relation to children, was unhelpful as adults were seen as a highly affected group. Advocacy stakeholders said that the emphasis on downstream policies targeting personal responsibility and individual action could result in further stigmatisation of those living with obesity. They felt that these were policies that would benefit the population as a whole and should be positioned as such to avoid stigmatising people living with overweight or obesity.

*‘It's not really obesity specific, it's interventions that could benefit people right across the population, it's not about obesity. So, yeah, reducing our, you know, sugar drink intake or not overconsuming in certain areas that's going to benefit everybody and it's going to benefit many people who are without obesity.’ (S34, policy)*

##### Theme 1.5: the food and drink industry already play a role in helping the UK population to eat more ‘healthily’

The most significant divergent view arising came from commercial interviewees, who emphasised that they already play active roles in improving population health through product development and the formulation of ‘healthy’ options.

*‘And if you don’t see the return on investment because you don’t see the ability to take that to market and succeed with it, the work simply won’t happen. And a consequence of that will be obviously kind of a reduction in innovation but also kind of a reduction in choice for consumers.’ (S28 commercial)*

They noted that this is an expensive process and further marketing restrictions would be a disincentive for such investment, with negative consequences for consumers.

#### Views on individual policies included in the July 2020 announcement

Interviewees were asked to consider the likely impact and feasibility of each of the policies contained in the July 2020 announcement (Table 1). Responses were categorised as either supportive or opposing arguments and by stakeholder type (Appendix C).

##### Theme 1.6: most supported policy proposals

The in-store restriction of HFSS promotions by place and price as well as a total ban on HFSS marketing were the policies most supported by policy and advocacy stakeholders with close to half of the interviewees in those groups supporting each policy, and 10–20% of commercial stakeholders supporting them as well. This was particularly true for restrictions by place which was deemed to have the most potential to impact on purchasing behaviour and reduce pester power.

*‘I think anything that restricts multibuys on HFSS and, you know, the end of aisle placement and all of those things is a good idea, because we know that it encourages people to buy more…want to buy more impulsively.’ (S10 policy)*

There was general agreement that these policies would be relatively straightforward to implement, and potentially supported by the retail sector where progressive retailers were already taking the initiative and would welcome the introduction of a level playing field. It was deemed important to be clear about how these policies might be applied, both in terms of which retail outlets (including non-food outlets) and which products they would be applied to.

A potential regressive negative consequence of restricting price promotions was discussed by all interviewees, particularly commercial stakeholders. All stakeholders highlighted that many families depend on price promotions to feed their families and for some the purchasing of HFSS products is one of the few ways they can treat their families.

*‘The multi-buy offers will impact families with less means and larger families, so yeah, if they are still going to buy their two bottles of Coke or whatever, then yes, they will have less money in their pocket.’ (S33 advocacy)*

This was thought to be particularly significant in the context of COVID-19 as parents’ financial challenges were heightened and particularly for those with large families who would still buy multi-packs of products.

##### Theme 1.7: most opposed policy proposals

Further advertising restrictions on TV and online received the most opposing arguments, from approximately half of policy and commercial stakeholders and one third of advocacy stakeholders, who cited difficulty in implementation as their reasoning. The 9 pm watershed was perceived as straightforward to implement on broadcast TV, where existing legislation and definitions of HFSS could be modified. However, all types of stakeholders agreed it would have limited impact on exposure as few young people consume entertainment in such a traditional manner.

*‘I just think that there's an argument to say that we're so far beyond what the 9:00 pm watershed actually means that I'm not sure that it's still contemporary or relevant. I’m not sure it will be as effective as people think.’ (S24 policy)*

Restricting online HFSS advertising was considered likely to be more effective, but far harder to achieve due to the complexity of the online environment. Stakeholders were unclear how the online environment, including social media, influencer marketing and cross border activities, could be defined and regulated.

##### Theme 1.8: policies with mixed responses

Other policies in the Government’s July 2020 Obesity Strategy received a more mixed response with labelling of all kinds (front-of-pack, out-of-home and calorie labelling on alcoholic drinks) deemed the most likely to do harm to some populations such as those living with weight stigma or disordered eating.

*‘A key part of getting people to combat and manage their disordered eating is about preventing food from being seen as taboo and it’s all about helping them to, sort of, revert to a place where they can choose to eat any food.’ (S29 advocacy)*

While improving the system for front-of-pack nutritional labelling was thought to have some potential in terms of helping with public education and prompting reformulation of processed foods, it was not seen as a high priority for any stakeholder group. All stakeholder types agreed that the measure would have questionable impact as it would rely on individuals changing their behaviour in response to the information provided. Policy stakeholders also said that if any new system were to be effective it would need to be mandatory, and commercial stakeholders agreed it would be helpful to have a level playing field in this respect.

##### Theme 1.9: defining ‘healthy’ and ‘unhealthy’ foods

A common theme underlying all discussions on the UK Government’s July 2020 Obesity strategy was the importance of having a clear definition of which foods these policies should apply to. All stakeholder types noted that it is unhelpful to have a binary system leading to a suggestion of ‘healthy’ and ‘unhealthy’ products as this is an over-simplification, can result in misconceptions about food, and does not help individuals understand how to have an overall balanced diet. The current UK nutrient profiling model (NPM) was thought to be useful in that it is well understood by industry and could be applied consistently across all policies. However, it was noted that the model was designed for a specific purpose (regulation of TV advertising to children) and although it is currently under review it would still have some weaknesses if applied more widely. For example, policy stakeholders noted that its product nutrient focus does not deal with brand advertising, and it can lead to unhelpful reformulation to meet specific nutrient targets. They noted that ‘lower fat’ and ‘lower sugar’ products were not necessarily ‘healthy’ options.

*‘With the current nutrient profiling model, for example, all industry really needs to do in order to negate the advertising restrictions around that, is bring it just under the threshold levels of that nutrient profiling model, which actually can still be pretty high for levels of sugar, fat and salt.’ (S02 policy)*

#### RQ 2: What other policy areas are important to consider when seeking to reduce obesity at a population level?

The mixed views on the July 2020 Obesity Strategy illustrate the fact that many stakeholders considered other policy priorities more important in tackling obesity, both specifically in relation to the marketing of HFSS foods and more broadly. Five key themes emerged from these wider discussions.

##### Theme 2.1: financial levers are potentially the most powerful, but they should be used with extreme caution

While most of the policy and advocacy stakeholders discussed the potential of using price to discourage consumption of ‘unhealthy’ foods, they universally highlighted the need to balance this with reducing the price of ‘healthy’ foods to avoid regressivity, particularly in the context of COVID-19. The use of subsidies was frequently cited as a means of achieving this, though some felt that rebalancing of VAT could be an option.

*‘It’s a regressive policy and it’s going to impact the poorest in society the most and that seems really unfair, and I think doing VAT where it’s charged on unhealthy food, but not healthy food would begin to address that.’ (S02 policy)*

Again, the importance of defining which foods should be covered by such policies was highlighted.

The soft drinks industry levy (SDIL) was cited as being particularly successful because sugary drinks are a discretionary product and relatively straightforward to reformulate, but participants suggested that this would be more challenging to achieve with other foods and drinks.

Commercial stakeholders were also cautious about using financial levers to raise prices of ‘unhealthy’ foods, but rather than counterbalancing this with subsidies they were more likely to suggest policy priorities that could tackle societal level drivers of obesity, such as inequalities.

*‘I don’t think we should be increasing the cost of food in this country for people that don’t necessarily always have the ability to make the choices that we would all wish to make.’ (S11 commercial)*

##### Theme 2.2: reformulation does not necessarily result in ‘healthy’ products

Reformulation was a key discussion point for all types of stakeholders. However, a note of caution was sounded by some policy and advocacy stakeholders concerning an overreliance on processed foods, and that existing reformulation programmes simply result in slightly less ‘unhealthy’ products rather than encouraging healthy eating practices.

*‘The big problem is consumption of ultra-processed food, and you can’t ignore it, it’s there, but we’re kind of backing that industry by saying, oh, it’s great, they’ve reformulated, you’ve got lower fats. So, is it really healthy to be encouraging and promoting these ultra-processed foods?’ (S03 policy)*

However, others observed that the reality of life is that people rely on processed foods and that it is therefore important to reduce their impact on health.

Commercial stakeholders emphasised that they already invest in extensive voluntary reformulation programmes in response to consumer preferences, particularly the move towards plant-based foods, and that marketing restrictions could disrupt this investment. However, policy stakeholders noted that existing voluntary reformulation programmes were having limited success, with some categories faring better than others in the recent Public Health England progress report [29].

##### Theme 2.3: to be effective, any HFSS advertising restrictions need to be comprehensive and complete across all venues, media and platforms

Policy and advocacy stakeholders were concerned about the potential balloon effect of marketing restrictions, particularly for advertising, where regulation of one form of advertising may simply drive additional investment in another form. They highlighted that for any advertising restrictions to be effective they need to be comprehensive, covering all possible outlets including: all advertising spaces in the physical environment, workplaces, hospitals, public and sporting venues, all media, and digital platforms. However, they recognised that this would be difficult to achieve and would meet significant resistance. Sporting venues were deemed particularly important in the context of COVID-19, with more sport being broadcast on TV and digital outlets and therefore seen by children and young people. Commercial stakeholders wanted to ensure that any policy in this area would be proportionate.

*‘Restricting marketing across all media, public venues and publicly funded events, that sounds huge as one single policy initiative to me. I wouldn’t be able to quantify what that would mean. But again, it comes down to, you know, what’s a proportionate approach.’ (S04 commercial)*

##### Theme 2.4: early years and preconception should be a key policy focus

A policy priority that went beyond restricting the marketing of HFSS foods and was raised by all three stakeholder types, but predominantly policy and advocacy stakeholders, was the need to improve nutrition for children and in the early years.

*‘I think that it would be really important to make sure that early years have enough support. It's a really, really important period of time that we can have quite a big impact in terms of food preferences and food habits.’ (S18 policy)*

The school setting was viewed as an important environment, but stakeholders argued that policies should also apply to preconception and maternal nutrition as well as the infant and complementary feeding stages.

In relation to school meals, advocacy stakeholders were particularly concerned with the failure to implement and monitor existing nutritional standards. One stakeholder highlighted that the policy objective should not be to provide more free school meals, but rather to eliminate the need for free school meals by tackling inequalities.

*‘…because you’ve allowed more poorer children to continue to live in poverty but get fed at school. … by 2030 to have no children on free school meals. That should be the policy’ (S35 advocacy)*

##### Theme 2.5: going beyond further restricting the marketing of HFSS products will be essential to tackle the underlying drivers of obesity

As highlighted by initial reactions to the July 2020 Obesity Strategy, stakeholders from all groups suggested that a focus on HFSS marketing restrictions was potentially a missed opportunity in that it failed to address the societal-level drivers of obesity. Policy stakeholders in particular commented that it would be more important to develop policies that promote systemic change, address the role that ‘unhealthy’ foods play in society, change social norms and address inequalities.

*‘This is not even a food policy because I think this is just the nature of the way that we live now, but to try and get people back into families eating together around the food table. I think getting back into a more positive food culture.’ (S09 policy)*

#### RQ3: how might Government policy improve the availability and promotion of ‘healthy’ options?

Four common themes arose from the discussions on what the UK Government could do to improve the consumption of a well-balanced diet at the population level, and thus reduce the prevalence of obesity.

##### Theme 3.1: it would be better to focus on making the healthy option the easy option and encouraging healthy lifestyles

All stakeholder types advocated balancing any further HFSS marketing restrictions with policies that would promote healthy lifestyles, including encouraging physical activity and nutritional guidance. They considered the availability and price of healthy options to be crucial. The importance of public health information on what constitutes a healthy diet was viewed as critical to underpin the effectiveness of any obesity strategy.

*‘They [Canada] have in their equivalent of the Eatwell Guide that people should eat more whole foods and in the associated blurb it says, if you have the choice, choose home prepared food over processed food. I think Brazil goes even further and says avoid ultra-processed food.’ (S01 policy)*

Although not necessarily the most powerful lever for eating behaviour change, effective public health campaigns were viewed as an important supportive component, specifically that they needed to be culturally relevant. Canada and Brazil were cited as good examples of countries with nutritional guidance that covers the social aspects of eating together and emphasises whole foods rather than individual nutrients.

##### Theme 3.2: an effective obesity strategy needs to work at every level in society

All three stakeholder types also stressed that no one policy was likely to work effectively in isolation, and that there needed to be multiple policies working in harmony.

*‘I think it’s national government set the ambition and the framework, local authority is empowered and funded to be able to get into where the issues are, and then the individual through their environment being empowered and then enabled to make the choices that they should make.’ (S11 commercial)*

They stressed the importance of having a strategy and policies that cover all levels of society from: supporting individual understanding; action in educational and employment settings; promoting a food environment that makes the ‘healthy’ option the easy option; the NHS including weight management services; local and national government policies.

##### Theme 3.3: make the most of opportunities to work in partnership with industry

Commercial stakeholders highlighted the fact they are already investing in strategies to promote healthy lifestyles, primarily in response to consumer demand. This included: reformulation programmes to remove sugar and fat and increase plant-based products; investment in TV adverts to promote vegetables to children; removal of cartoon characters from HFSS products; removal of HFSS products from key selling locations; and trialling nudge techniques such as menu cards in retail locations.

These stakeholders observed that NGOs had worked with industry on some of these campaigns, and suggested that Government could learn from these programmes, rather than introducing blanket restrictions which they saw as less effective in influencing eating behaviour.

*‘I think all these kinds of things help and it would be good, I think, to see government taking more interest and embracing a holistic approach which brings together and talks about all these things, and gets industry and NGOs and charities and everybody, all stakeholders, working together in a kind of constructive way, instead of arguing about policies. I think that would be really helpful.’ (S04 commercial)*

## Discussion

The role and relative importance of HFSS marketing restrictions in obesity policy was largely supported by all three groups of stakeholders with commercial stakeholders advocating for more cross-sector approaches and collaboration between government and industry. The three groups shared a frustration with the policy process, arguing that its focus on marketing will not contribute to efforts to address the underlying drivers of obesity such as socioeconomic position, cultural norms, and the physical food environment. Their frustration and push for proactive government action is supported by the fact that policy proposals in the past have not been consistently acted upon, evaluated, or cross-referenced when creating new proposals [2]. Conducting interviews in September-December 2020, shortly after the publication of the Obesity strategy and amid a series of COVID-19 lockdowns before vaccines became widely available in the UK allowed us to engage with experts during a particularly tense time when motivation to address obesity was being prioritised. The pandemic context may have encouraged the stronger government action outlined in the proposal and allowed for the pleasant surprise and cautious optimism felt among stakeholders. However, recent delays in implementation of the outlined policies point to a repeat of past lapses in government efforts to combat obesity in the UK [2, 21].

While all groups of stakeholders advocated for policies beyond marketing restrictions, they also expressed a belief that restrictions on HFSS marketing do have a role to play in reducing overweight and obesity, if implemented properly. Restrictions on the marketing of HFSS products in London and Chile have shown reductions in purchasing of such products [27–29] and several studies have shown the benefits of implementing restrictions on HFSS advertising for children and young people [30–33]. The protection of children and young people from HFSS food marketing is particularly important given the continued rise in obesity in this age group, the limited ability of young children to differentiate between advertisements and entertainment, and the potential effects throughout the life course of a diet high in HFSS foods [34, 35]. Further research on the long-term impacts of marketing restrictions on HFSS foods and implications for the health of children and adults is still needed.

The more interventionist nature of the July 2020 proposal sends a strong message regarding the Government’s intentions to reduce obesity in the UK, with some policies receiving more support than others from stakeholders. Restrictions on price and place were largely supported by all three groups and previous research has shown that retailers that do not place ‘less-healthy’ foods near the checkout area sell fewer of these items [16]. The measures outlined to restrict the marketing of HFSS foods are simpler to implement for certain product classifications such as sugar-sweetened beverages, but there is continued debate about how best to classify different foods and beverages [36, 37]. Policy stakeholders noted that product reformulation is not the answer as it can lead to changes to meet specific nutrient thresholds without improving the overall ‘healthiness’ of the food, while commercial stakeholders argued that the ongoing reformulation and creation of new products to meet consumer demand for ‘healthier’ foods is beneficial. Recent research demonstrates an association between ultra-processed food consumption and multiple adverse health outcomes [38, 39], further supporting the position that ‘lower fat’ or ‘lower sugar’ products are not necessarily ‘healthier’ if they are ultra-processed. In general, the positionality of participants was reflected in their responses, with commercial stakeholders asserting that private companies are willing to take action to improve consumers’ health, policy stakeholders arguing for more interventionist policies, and advocacy stakeholders bringing attention to potential effects on specific groups of people.

Stakeholders from all three groups expressed a belief that measures to reduce obesity should be comprehensive and complete to be effective, which aligns with research that advocates for multiple types of policy to target the underlying causes of obesity such as income inequality, social norms, and the general food environment [27, 40]. There has been limited impact on obesity prevalence in the last 30 years in the UK, with rates continuing to climb despite the regular publication of policy proposals aiming to reduce them [2]. As Theis and White point out, these proposals are often not implemented in full and tend to focus on individual agency and behavioural change rather than looking at external influences. Policies restricting HFSS marketing, while thought to be progressive [27] do not go far enough in shifting societal norms, eating behaviours (such as promoting whole foods or eating as a family), food pricing, inequalities, and poverty. Potentially regressive policies, such as restricting volume promotions, should be paralleled with measures to facilitate the availability and affordability of alternatives. Additionally, the proposed restrictions on TV advertisements may have a smaller impact than anticipated due to the shift in media consumption to online platforms such as social media and video streaming services which are more complex and therefore harder to regulate [41]. In order to robustly implement this policy, a comprehensive 9 pm watershed across all media would be needed [42]. Research on exposure to HFSS advertisements online and how best to limit it is an important area for future research. In general, structural measures are more effective and equitable than those that focus on individual agency [2] and further efforts to promote systemic change and make healthier choices easier choices are necessary if significant progress is to be made on obesity rates in the UK without exacerbating inequalities.

This was a qualitative study involving semi-structured interviews conducted over Microsoft Teams with 42 total stakeholders across the UK: 19 policy, 10 commercial and 13 advocacy actors. The research team purposively identified and directly invited stakeholders with expertise in obesity policy and the development and use of HFSS marketing restrictions. Lay stakeholders with experience of working with the stigma community, children, young people and those living with inequalities were also specifically targeted. A key strength of the study is that the topicality of the subject matter was helpful in securing a high level of engagement from invited participants. Pivoting the methods to online rather than face-to-face or telephone interviews also strengthened the study substantially by increasing participation.

However, some limitations should be noted. One limitation of the study was that the number of stakeholders in each sub-category was relatively small. This meant that we could not represent views by stakeholder sub-type as to do so would risk divulging the identity of individual participants based on specifics of their professional activities. Additionally, qualitative methods inherently cannot provide representative evidence of the distribution of different perspectives within a population. However, the use of purposive sampling methods permitted a diverse breadth of relevant perspectives to be captured, and for fringe or unanticipated perspectives to emerge. Our use of qualitative thematic analysis brought with it the question of trustworthiness inherent to this methodology, which we attempted to mitigate through careful consideration in the recruitment, data collection and analysis processes. Finally, more policy stakeholders (*n* = 19) agreed to participate compared to commercial stakeholders (*n* = 10) or lay stakeholders (*n* = 13), which meant the voice of policy stakeholders may have been more dominant in the data. However, the NVivo analysis allowed frequency of views on any subject to be calculated as a percentage of the number of stakeholders of that type, thus making the data comparable.

## Conclusion

The UK Government’s proposed policies to address obesity were met with mixed reactions from the policy, advocacy and commercial stakeholders interviewed, with general agreement that tackling obesity is important and should be approached from multiple angles and differing views on how best to accomplish this goal. Future research should evaluate the effectiveness of the policies that are implemented and continually review stakeholder responses to their rollout.

### Supplementary Information

**Additional file 1: Appendix A.**

**Additional file 2: Appendix B.**

**Additional file 3: Appendix C.**

**Additional file 4: Appendix D.**

## Data Availability

Interview transcripts cannot be shared due to confidentiality. All materials, including details about the search strategy and coding framework, are available in the Appendices.

## Abbreviations

CMO: Chief Medical Officer
CRUK: Cancer Research UK
HFSS: High in fat, sugar and/or salt
NPM: Nutrient profiling model
SDIL: Soft drinks industry levy
UK: United Kingdom
