# Supplementary material for: Expert views on high fat, salt and sugar food marketing policies to tackle obesity and improve dietary behaviours in the UK: a qualitative study
Source: BMC Public Health. 2023 Oct 9;23:1951. doi: 10.1186/s12889-023-16821-2 (PMC10561510; doi:10.1186/s12889-023-16821-2)
Supplement: Supplementary file 1 — Additional file 1: Appendix A. [file 12889_2023_16821_MOESM1_ESM.docx]

**Appendix A: Interview topic guides**

**Topic guide 1: Policy expert interviews**

**Introductory section**

- Hello, I am a research assistant from the University of Glasgow with an interest in health policy. You’ll be aware from the information sheet that we’re doing research into how different groups of people feel about policies for regulating the marketing of food and drink high in fat, salt or sugar. You may already be aware of the UK Government announcement on this in July this year, which was apparently prompted by the association between obesity and worse outcomes from COVID-19.
- Reiterate key points from consent form, check verbally that participant has read the information sheet and is happy to participate and be recorded.
- Describe study and scope of questions. Set in the context of the recently announced UK obesity strategy in the light of COVID-19. Explain the broad definition of marketing used, explaining the four Ps of the marketing mix.
- Are you Ok to continue / happy for me to record?

**Background Question:** Can you describe your professional role, and how it relates to policy restricting the marketing of unhealthy food?

**Section 1: Current policy responses** – announced by the UK Government as part of the obesity strategy in July 2020, and in response to COVID-19 ‘Tackling obesity: Empowering adults and children to live healthier lives’.

For commercial stakeholders:

1. Does your organisation have an ambition to reduce marketing of unhealthy foods, and to reduce childhood obesity?
   - - Prompt: If yes, what barriers does your organisation face?
     - Prompt: What would help to overcome the barriers?
2. Who has a role to play in reducing people’s consumption of unhealthy foods?
   - - Prompt: What is the role of Government vs Industry vs Individual responsibility?
     - Prompt: When should Government step in? Level of ‘market failure’

For lay stakeholders:

1. Do you think government policy has a role to play in reducing population consumption of unhealthy food and drink?
2. Could policies to restrict marketing of unhealthy food and drink have unintended consequences for the public?
   - - Prompt: Is there a risk of policy worsening the stigma that obese children and adults experience?
     - Prompt: Is there a risk of policy worsening problems of poverty and social inequality in children and adults?
     - Prompt: How can policymakers avoid making these problems worse, or help solve these problems?

Now we’re going to talk about some different government policy options to further restrict the marketing of food high in fat, salt or sugar, as part of their strategy to tackle obesity – particularly in children and young people.

1. If you look at Sheet A, you’ll see a set of policies announced by the UK Government in July 2020 – either for implementation or further consultation. What is your reaction to this set of policies as a strategy for tackling obesity?
   - Prompt: Do any of them stand out as particularly important to you?
   - Prompt: How easy will it/they be to implement – Barriers? Facilitators?
   - Prompt: What might the impacts be, particularly for children and young people?
   - Prompt: How might we expect the food and drink industry to respond to this policy?
   - Prompt for policy stakeholders:
     - - What legislation is needed? How difficult?
       - Industry narrative? Lobbying tactics? Loopholes?
   - Prompt for commercial stakeholders*:*
     - - Might there be any unintended consequences for consumers and the market?
   - Prompt for lay stakeholders:
     - - Might there be any unintended consequences in terms of stigma or poverty?
       - How can the movements of body positivity/ reducing weight stigma and the goal of reducing obesity for disease prevention be married up?
       - How do we continue to implement policies that help reduce obesity but do so in a way that does not worsen stigma?
       - Do you think food manufacturers and retailers would support this strategy?

Additional prompts (specifically from CRUK):

- - What method would you prefer the Government used for defining which food and drink was in scope and thus couldn’t be advertised/promoted?
  - Would you prefer the same approach used for both promotions and marketing restrictions, or could they be different?
  - Are there any types of TV channels / online sites / platforms / retail locations which you think should be excluded from restrictions, or would you favour a blanket ban?

1. Do any seem like a bad/less important idea? Why?
   - Prompt: Effectiveness? Implementation issues? Unintended consequences?
   - Prompt for lay stakeholders:
     - - What is wrong with the idea?
       - Unintended consequences? Stigma or Poverty? People with eating disorders?
2. What about [name a policy that has not been discussed, in a quadrant that has not been discussed]? Do you think that is a worthwhile policy?
   - Prompt: How easy will it/they be to implement – Barriers? Facilitators?
   - Prompt: What legislation is needed? How difficult?
   - Prompt: What might the impacts be, particularly for children and young people?
   - Prompt: How might we expect the food and drink industry to respond to this policy?
   - Prompt for policy stakeholders:
     - - Industry narrative? Lobbying tactics? Loopholes?
   - Prompt for commercial stakeholders:
     - - How would the food and drink industry be likely to respond to this strategy?
       - Might there be any unintended consequences for consumers and the market?
   - Prompt for lay stakeholders:
     - - Might there be any unintended consequences in terms of stigma or poverty?
       - How can the movements of body positivity/ reducing weight stigma and the goal of reducing obesity for disease prevention be married up?
       - How do we continue to implement policies that help reduce obesity but do so in a way that does not worsen stigma?
       - Do you think food manufacturers and retailers would support this strategy?
3. Do you think any of these policies stand out as being particularly valuable for tackling obesity in children. Why?
4. Thinking about place, product, promotion and price, do you think any of these are particularly important to focus on?
   - Prompt for policy stakeholders: What do you think about product not being covered so much by the policies currently under consideration?

**Section 2: Other potential policy options**

Now we’re going to talk about some potential other policies. Looking at Sheet B, each of these policies were proposed last year in a report on solving childhood obesity by the outgoing Chief Medical Officer for England.

1. Which of these policy options would you say are the top three priorities and why? Prompts for each policy mentioned:
   - Prompt: How feasible do you think this policy would be in practice?
   - Prompt: How easy will it/they be to implement – Barriers? Facilitators?
   - Prompt: What legislation will be needed? How difficult?
   - Prompt: What might the impacts be on children and young people?
   - Prompt for policy stakeholders:
     - - How might we expect the food and drink industry to respond to this policy?
       - Industry narrative? Lobbying tactics? Loopholes?
   - Prompt for commercial stakeholders:
     - - How would the food and drink industry be likely to respond to this strategy?
       - Might there be any unintended consequences for consumers and the market?
   - Prompt for lay stakeholders:
     - - Might there be any unintended consequences in terms of stigma or poverty?
       - How can the movements of body positivity/ reducing weight stigma and the goal of reducing obesity for disease prevention be married up?
       - How do we continue to implement policies that help reduce obesity but do so in a way that does not worsen stigma?
       - Do you think food manufacturers and retailers would support this strategy?
2. Do any of these policies seem like bad ideas, or less important ideas? Why?
   - Prompt: Effectiveness? Implementation issues? Unintended consequences?
   - Prompt for lay stakeholders:
     - What is wrong with the idea?
     - Stigma or Poverty? People with eating disorders?
3. What about [name a policy that has not been discussed, in a quadrant that has not been discussed]? Do you think that is a worthwhile policy?
   - Prompt: How easy will it/they be to implement – Barriers? Facilitators?
   - Prompt: What legislation will be needed? How difficult?
   - Prompt: What might the impacts be, particularly for children and young people?
   - Prompt for policy stakeholders:
     - How might we expect the food and drink industry to respond to this policy?
     - Industry narrative? Lobbying tactics? Loopholes?
   - Prompt for commercial stakeholders:
     - How would the food and drink industry be likely to respond to this strategy?
     - Might there be any unintended consequences for consumers and the market?
   - Prompt for lay stakeholders:
     - Might there be any unintended consequences in terms of stigma or poverty?
     - How can the movements of body positivity/ reducing weight stigma and the goal of reducing obesity for disease prevention be married up?
     - How do we continue to implement policies that help reduce obesity but do so in a way that does not worsen stigma?
     - Do you think food manufacturers and retailers would support this strategy?
4. Are there any other good ideas for policies for tackling obesity, both for children and in the general population, that we haven’t discussed yet?

**Section 3: Key policy priorities and improving the availability and promotion of healthy products**

1. Having discussed a variety of different policy approaches to restricting the marketing of HFSS foods, is there an area of policy action that you think is particularly important to pursue, that the Government is not already considering?
   - Prompt: If you could progress one policy from Sheet B, which would it be?
2. If you could do anything to tackle childhood obesity, what would it be? [if not already discussed]
   - Prompt: Are there examples from other countries where governments are ‘getting it right’?
3. In addition to policies to restrict the marketing of unhealthy products, do you have any ideas for policies that would improve the availability and promotion of healthy food and drinks?

4a. For lay stakeholders: Is there anything important that researchers and policymakers need to consider when exploring options for tackling obesity? (i.e., stigma, poverty, eating disorders)

4b. For commercial stakeholders: What do we need to know more about to take effective steps to tackle obesity? Is there something that research should be focusing on?

4c. For policy and commercial stakeholders: Are you aware of any international examples of policies that combine positive approaches with restrictions?

4d. For policy stakeholders: What are the key gaps in knowledge where evidence could help to inform policy related to marketing of HFSS food and drink?

- - Prompt: Why do you think it’s important to reduce obesity?
  - Prompt: Based on what evidence?

1. Do you think the context of COVID-19 makes any difference to how we should approach tackling obesity in the UK? [If not already discussed]
   - Prompt: stronger Government action? Increase restrictions on industry?
