# Supplementary figures and images for: Expert views on high fat, salt and sugar food marketing policies to tackle obesity and improve dietary behaviours in the UK: a qualitative study
Source: BMC Public Health. 2023 Oct 9;23:1951. doi: 10.1186/s12889-023-16821-2 (PMC10561510; doi:10.1186/s12889-023-16821-2)

**Appendix B: Visual prompts for interviews**

**
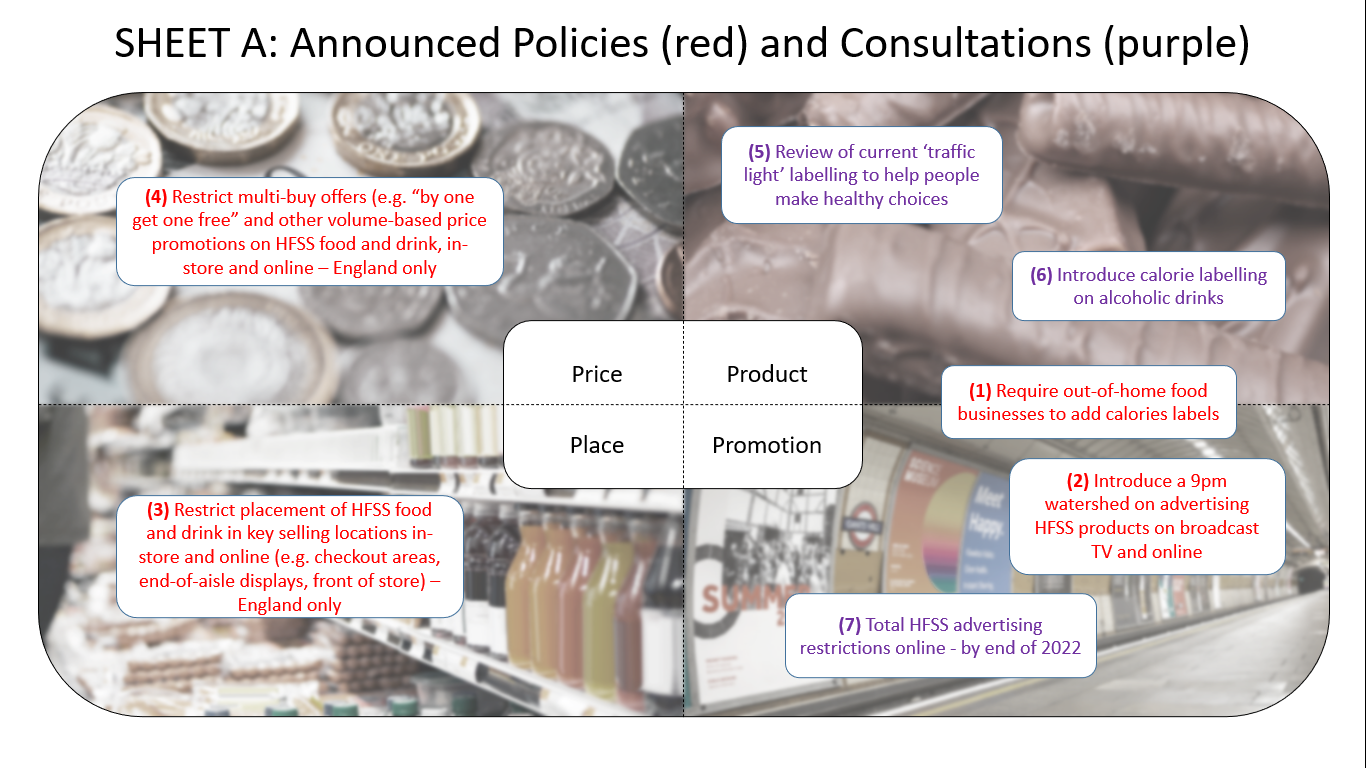
**


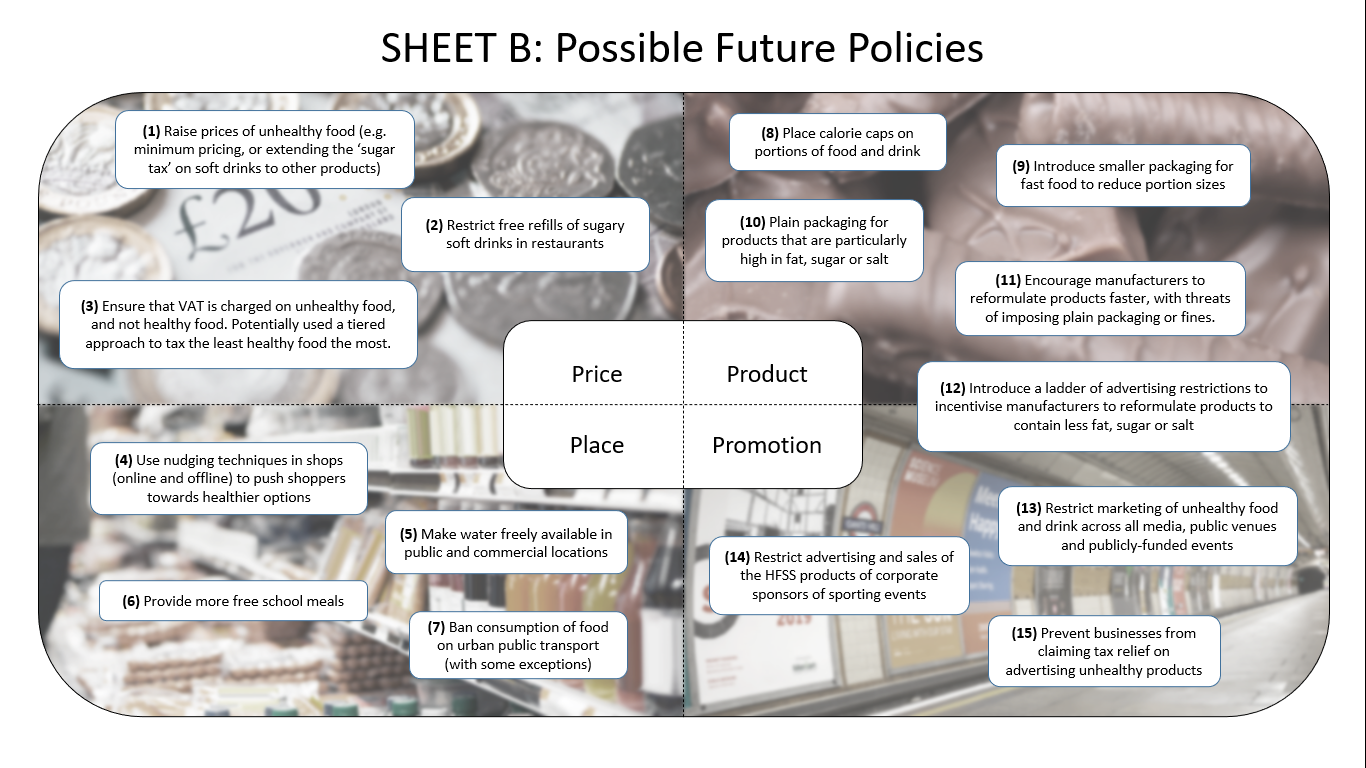

Supplement: Supplementary file 2 — Additional file 2: Appendix B. [file 12889_2023_16821_MOESM2_ESM.docx]
