# Supplementary material for: Expert views on high fat, salt and sugar food marketing policies to tackle obesity and improve dietary behaviours in the UK: a qualitative study
Source: BMC Public Health. 2023 Oct 9;23:1951. doi: 10.1186/s12889-023-16821-2 (PMC10561510; doi:10.1186/s12889-023-16821-2)
Supplement: Supplementary file 3 — Additional file 3: Appendix C. [file 12889_2023_16821_MOESM3_ESM.docx]

**Appendix C Tables**

Table C1: Stakeholder attitudes

|  | **A: Stakeholder: Stakeholder type = 1.0 Policy stakeholder** | **B: Stakeholder: Stakeholder type = 2.0 Commercial stakeholder** | **C: Stakeholder: Stakeholder type = 3.0 Lay stakeholder** |
| --- | --- | --- | --- |
| **1 : Mixed feelings** | 17 | 4 | 10 |
| **2 : Mostly negative** | 10 | 8 | 7 |
| **3 : Mostly positive** | 16 | 3 | 10 |
| **4 : Neutral** | 8 | 2 | 4 |

Table C2: Supportive arguments

|  | **A: Stakeholder: Stakeholder type = 1.0 Policy stakeholder** | **B: Stakeholder: Stakeholder type = 2.0 Commercial stakeholder** | **C: Stakeholder: Stakeholder type = 3.0 Lay stakeholder** |
| --- | --- | --- | --- |
| **1 : Helpful to have clear nutritional information** | 8 | 2 | 6 |
| **2 : Helps create a level playing field** | 5 | 3 | 4 |
| **3 : Is effective or important** | 15 | 2 | 9 |
| **4 : Is feasible or easy to do** | 8 | 1 | 2 |
| **5 : May work by prompting reformulation** | 4 | 0 | 2 |
| **6 : More important for adults** | 4 | 0 | 0 |
| **7 : Part of the picture** | 9 | 0 | 4 |
| **8 : Particularly for children and young people** | 6 | 1 | 8 |
| **9 : Supported by industry** | 4 | 2 | 1 |
| **10 : Supported by public** | 3 | 1 | 0 |

Table C3: Opposing arguments

|  | **A: Stakeholder: Stakeholder type = 1.0 Policy stakeholder** | **B: Stakeholder: Stakeholder type = 2.0 Commercial stakeholder** | **C: Stakeholder: Stakeholder type = 3.0 Lay stakeholder** |
| --- | --- | --- | --- |
| **1 : Devil is in the detail** | 12 | 4 | 3 |
| **2 : Difficult to implement** | 13 | 3 | 5 |
| **3: Disproportionate response** | 0 | 4 | 0 |
| **4 : Less relevant for children and young people** | 9 | 3 | 2 |
| **5 : Nanny state** | 0 | 2 | 1 |
| **6 : Negative consequences** | 3 | 6 | 4 |
| **7 : No impact on obesity** | 2 | 4 | 0 |
| **8 : Online environment very difficult** | 11 | 5 | 5 |
| **9 : Only helpful to limited groups** | 0 | 0 | 3 |
| **10 : Potential for loopholes** | 6 | 0 | 1 |
| **11 : Potential to create stigma** | 3 | 1 | 7 |
| **12 : Questionable impact** | 12 | 7 | 6 |
| **13 : Regulatory burden** | 2 | 2 | 1 |
| **14 : Requires individual action** | 1 | 2 | 2 |
| **15 : Unpopular with industry** | 7 | 4 | 3 |
| **16 : Unpopular with public** | 3 | 0 | 0 |
