# Supplementary material for: Expert views on high fat, salt and sugar food marketing policies to tackle obesity and improve dietary behaviours in the UK: a qualitative study
Source: BMC Public Health. 2023 Oct 9;23:1951. doi: 10.1186/s12889-023-16821-2 (PMC10561510; doi:10.1186/s12889-023-16821-2)
Supplement: Supplementary file 4 — Additional file 4: Appendix D. [file 12889_2023_16821_MOESM4_ESM.docx]

**Appendix D: Coding Framework**

**Structural Codes**

| **Codes** | **Themes** |
| --- | --- |
| Initial reactions to July 2020 plan |  |
| Initial reactions to CMO report |  |
| Views on defining HFSS foods |  |
|  | Better to define degree of processing |
|  | Make simpler e.g. use categories such as discretionary foods |
|  | Needs to be different for different policies |
|  | No such thing as ‘healthy’ and ‘unhealthy’ |
|  | NPM doesn’t deal with brand advertising |
|  | NPM is robust and founded on evidence |
|  | NPM produces anomalies |
|  | NPM promotes unhelpful reformulation |
|  | NPM provides consistency but may not be perfect |
|  | WHO method is better |
| Industry responses |  |
|  | Speeds up something we were doing anyway |
|  | Subsegments react differently |
|  | Tend to react initially and then quieten down |
|  | Typical arguments |
|  | Unfair in the COVID context |
|  | Won’t work need to work together |
| How to promote healthy options |  |
| Evidence gaps |  |
| Other policy priorities |  |
|  | Coherent strategy—no one priority |
|  | Encourage employer responsibility |
|  | Encourage physical activity |
|  | Focus on whole diet not HFSS |
|  | Link to environmental impacts |
|  | Make it culturally relevant |
|  | Make the ‘healthy’ option the easy option |
|  | Need systemic change |
|  | Need to address the role they play in society |
|  | Need to change social norms |
|  | Need to tackle inequalities |
|  | Need to tackle whole population not just children |
|  | Partnership with industry |
|  | Public health information campaigns |
|  | Schools and early years |
|  | Weight management support |
| COVID context |  |
| Brexit context |  |
| Inequalities |  |
| International examples |  |
| Framing |  |
| Stigma |  |
| Unintended consequences |  |
| Scotland specific |  |

**Attitudinal Codes—policy specific**

| **Codes** | **Themes** |
| --- | --- |
| Supportive arguments |  |
|  | Supported by public |
|  | Supported by industry |
|  | Particularly for children and young people |
|  | Part of the picture |
|  | More important for adults |
|  | May work by prompting reformulation |
|  | Is feasible or easy to do |
|  | Is effective or important |
|  | Helps create a level playing field |
|  | Helpful to have clear nutritional information |
| Opposing arguments |  |
|  | Unpopular with public |
|  | Unpopular with industry |
|  | Requires individual action |
|  | Regulatory burden |
|  | Questionable impact |
|  | Potential to create stigma |
|  | Potential for loopholes |
|  | Only helpful to limited group |
|  | Online environment very difficult |
|  | No impact on obesity |
|  | Negative consequences |
|  | Nanny state |
|  | Less relevant for children and young people |
|  | Disproportionate response |
|  | Difficult to implement |
|  | Devil is in the detail |
| Neutral |  |
| Mostly positive |  |
| Mostly negative |  |
| Mixed feelings |  |

**Attitudinal codes—July announcement**

| **Codes** |
| --- |
| Pleasantly surprised |
| Disappointed—wrong policy focus |
| Disappointed—not enough |
| Cynical—seen it before |
| Cautiously optimistic |

**Attitudinal Codes—CMO report**

| **Codes** |
| --- |
| Wrong policy focus |
| Unfortunate |
| Potentially unworkable |
| Potential to have a significant impact |
| Positive but missing key elements |
| Another positive step |
